# Infection with Carbapenem-resistant Hypervirulent *Klebsiella Pneumoniae*: clinical, virulence and molecular epidemiological characteristics

**DOI:** 10.1186/s13756-023-01331-y

**Published:** 2023-11-13

**Authors:** Linlin Li, Shan Li, Xianzhen Wei, Zhaolu Lu, Xue Qin, Meng Li

**Affiliations:** Key Laboratory of Clinical Laboratory Medicine of Guangxi Department of Education, Department of Clinical Laboratory, the First Affiliated Hospital of Guangxi Medical University, Nanning, China

**Keywords:** Carbapenem-resistant, Hypervirulent *Klebsiella Pneumonia*, Clinical characterization

## Abstract

**Background:**

Carbapenem-resistant hypervirulent *Klebsiella pneumoniae* (CR-hvKP) is gradually becoming the dominant nosocomial pathogens in the healthcare setting.

**Methods:**

A retrospective study was conducted on patients with CR-KP from July 2021 to May 2022 in a teaching hospital. We identified bacterial isolates, collected the clinical data, and performed antimicrobial susceptibility testing, hypermucoviscosity string test, antimicrobial and virulence-associated genotype, as well as multi-locus sequence typing. CR-hvKP was defined as the presence of some combination of *rmpA* and/or *rmpA2* with *iucA*, *iroB*, or *peg-344*. SPSS was used for data analysis. Univariate logistic regression analyses were used for risk factor and all statistically significant variables were included in the multivariate model. Statistical significance was taken to be P < 0.05.

**Results:**

A total of 69 non-duplicated CR-KP isolates were collected, 27 of which were CR-hvKP. Out of the 69 CR-KP strains under investigation, they were distributed across 14 distinct sequence types (STs), wherein ST11 exhibited the highest prevalence, constituting 65.2% (45/69) of the overall isolates. The principal carbapenemase genes identified encompassed *bla*_*kpc−2*_, *bla*_*NDM−1*_, and *bla*_*OXA−48*_, with *bla*_*kpc−2*_ prevailing as the predominant type, accounting for 73.9% (51/69). A total of 69 CR-KP strains showed high resistance to common clinical antibiotics, with the exception of ceftazidime/avibactam. The ST11 (P = 0.040), ST65 (P = 0.030) and *bla*_*kpc−2*_ ST11 clones (P = 0.010) were found to be highly related to hvKp. Regarding the host, tracheal intubation (P = 0.008), intracranial infection (P = 0.020) and neutrophil count (P = 0.049) were significantly higher in the patients with CR-hvKP. Multivariate analysis showed tracheal intubation to be an independent risk factor for CR-hvKP infection (P = 0.030, OR = 4.131). According to the clinical data we collected, tracheal intubation was performed mainly in the elderly with severe underlying diseases, which implied that CR-hvKP has become prevalent among elderly patients with comorbidities.

**Conclusions:**

The prevalence of CR-hvKP may be higher than expected in the healthcare setting. CR-hvKP is gradually becoming the dominant nosocomial pathogen, and its prevalence and treatment will be a major challenge. It is essential to enhance clinical awareness and management of CR-hvKP infection.

**Supplementary Information:**

The online version contains supplementary material available at 10.1186/s13756-023-01331-y.

## Introduction

*Klebsiella pneumoniae* (KP) is an increasingly critical hospital pathogen causing severe infection, including pneumonia, bacteremia, meningitis, liver abscess and urinary tract infection [1]. Over the past two decades, KP has evolved into two different evolutionary genetic lines: classical KP (cKP) and hypervirulent KP (hvKP) [2]. Early studies attributed a positive string test with a length > 5 mm as a hypermucoviscous phenotype, which is a traditional trait for hvKp strains. However, many studies do not agree with this hypermucoviscosity phenotype definition of hvKP since, on the one hand, not all hvKP strains are hypermucoviscous and, on the other, some cKP strains possess this characteristic [3, 4]. Thus, the use of a positive string test as the sole indicator of hvKp is insufficient.

Recently, multiple biomarkers, including the putative metabolite transporter (*peg-344*), salmochelin (*iroB*), siderophore aerobactin (*iucA*), regulator of mucoid phenotype A (*rmpA*) and regulator of mucoid phenotype A2 (*rmpA*2), have demonstrated > 0.95 diagnostic accuracy for identifying hvKP strains [5]. The genes *rmpA* and *rmpA2* were associated with the hypermucoviscous phenotype [6, 7], *iucA*, *iroB* and *peg-344* on virulence plasmids related to the hypervirulent (hv) phenotype of KP [8], which indicated that the use of a combination comprising *rmpA* and/or *rmpA2* with *iucA*, *iroB*, or *peg-344* to define hvKP would be more reliable.

Many studies have indicated that carbapenem-resistant KP (CR-KP) is associated with high morbidity and mortality, especially the hypervirulent strain (CR-hvKP) [9]. Mechanisms for the emergence of CR-hvKP can be succinctly delineated through two primary patterns: (i) the acquisition of a carbapenem-resistant phenotype by hypervirulent Klebsiella pneumoniae (hvKP) strains [10]; and (ii) the acquisition of a hypervirulent phenotype by carbapenem-resistant Klebsiella pneumoniae (CRKP) strains [11]. In recent years, the emergence of CR-hvKP has been continually and increasingly reported in China [12–14]. In addition, numerous studies have indicated that CR-hvKP can spread readily in clinical settings, causing fatal outbreaks, a propensity that has attracted worldwide attention [6, 15, 16]. Therefore, CR-hvKP is considered a serious threat to global health with the potential to be the next ‘superbug’ [17]. The studies referred to emphasize the importance of the ongoing surveillance of CR-hvKp infection and the need to understand the clinical characteristics, risk factors and molecular characteristics of this pathogen.

To date, there has been very little research on CR-hvKP in South China. Thus, for further investigation of the clinical characteristics, risk factors, molecular, prevalence and recent trend of CR-hvKP, we conducted a retrospective study in a teaching hospital in Nanning, South China, based on the newly validated CR-hvKP biomarkers *rmpA*, *rmpA2*, *iucA*, *iroB* and *peg-344* (i.e., the presence of a combination of the genes mentioned were used to classify hvKP).

## Materials and methods

### Bacterial isolates and identification

A total of 69 CR-KP non-duplicated isolates were collected consecutively from July 2021 to May 2022 at the First Affiliated Hospital of Guangxi Medical University in Nanning, China. CR-KP was defined as a clinical strain with resistance to carbapenems (including imipenem, meropenem and ertapenem) according to the breakpoints of the Clinical and Laboratory Standards Institute (CLSI) guidelines. All isolates were identified by the matrix-assisted laser desorption/ionization time-of-flight mass spectrometry system (MALDI-TOF/MS; BioMérieux, Lyons, France) or VITEK2 Compact system (BioMérieux, Marcy l’Etoile, France); the quality control strains used were *Pseudomonas aeruginosa* ATCC27853 and *Escherichia coli* ATCC25922 (National Center for Clinical Laboratories, Beijing, China). These strains were stored at − 80 °C for further study.

### Clinical data collection

The hospital’s electronic medical records were reviewed to collect all the clinical information of patients with positive CR-KP during the research period. The information collected included basic demographics (gender and age), underlying diseases, admission temperature, invasive procedures, surgery, antibiotic exposures, use of chemotherapy, admission to intensive care unit (ICU), previous hospitalizations, length of stay in hospital and outcomes.

### Antimicrobial susceptibility testing

Antibiotic susceptibility tests were performed for the isolates using the VITEK 2 Compact system or the disk-diffusion method. The results were interpreted as recommended by the CLSI (version 2021) and the European Committee on Antimicrobial Susceptibility Testing (EUCAST 2020) (http://www.eucast.org). In our study, the common clinical antibiotics found to have been used included ceftazidime/avibactam, levofloxacin, cefazolin, ceftriaxone, amoxicillin-clavulanic acid, piperacillin-tazobactam, cefoxitin, cefepime, aztreonam, ertapenem, imipenem, amikacin, gentamicin, tobramycin, ciprofloxacin, sulfamethoxazole, cefuroxime, cefperazone-sulbactam, meropenem, ceftazidime and piperacillin.

### String test

The string test was used for the identification of a hypermucoviscous phenotype as previously described [18]. In short, after growing KP on 5% sheep blood agar plates at 37 °C overnight, a standard bacteriological loop is used to stretch a ‘string’ of mucous from the bacterial colony. A positive test is indicated if the mucoviscous string is longer than 5 mm.

### DNA extraction

Genomic DNA was extracted from the CR-KP strains based on the instructions of the Biospin Bacteria Genomic DNA Extraction kit (Bioflux, Hangzhou, China). Finally, approximately 200 ul of the DNA solution was obtained to be used as a template for DNA reaction. The DNA was stored at − 20 °C for further research.

### Detection of virulence genes and carbapenemase genes

Virulence-associated plasmids such as pNTUH-K2044, pLVPK, and pLVPK-like harbour notable genetic markers including *peg-344, iroB, iucA, rmpA*, and *rmpA2* [6, 19, 20]. Measurement of these specific genes can serve as indicative measures for the presence of virulence-associated plasmids. In the context of our current investigation, the assessment of virulence plasmids was confined to the utilization of primers targeting *peg-344, iroB, iucA, rmpA*, and *rmpA2*. This approach was adopted purely for screening purposes in relation to the presence of virulence plasmids. Thus, the putative genes associated with virulence (*peg-344*, *iroB*, *iucA*, *rmpA* and *rmpA2*) and with carbapenem resistance (*KPC*, *NDM*, *IMP*, *VIM* and *OXA-48*) were detected by polymerase chain reaction (PCR) using specific primers as previously described (Table S1). [5, 21] Subsequently, the PCR products were visualised by agarose (1%) gel electrophoresis. The amplified positive PCR products were further confirmed by direct DNA sequencing (Sangon Biotech, Shanghai, China). Nucleotide sequences were compared by Basic Local Alignment Search Tool (BLAST) (http://blast.ncbi.nlm.nih.gov/Blast.cgi).

### Multi-locus sequence typing (MLST)

Seven housekeeping genes (*gapA, mdh, phoE, tonB, infB, pgi and rpoB*) were amplified by PCR according to the protocol (http://bigsdb.pasteur.fr/klebsiella/klebsiella.html) (Table S1). The PCR amplified products were sequenced (Sangon Biotech, Shanghai, China), and allelic profiling and sequence types (STs) determination were confirmed using abovementioned website.

### Statistical analysis

IBM statistical product and service solutions (SPSS) (version 25.0) was performed for data analysis. The measurement data were evaluated as mean ± standard deviations, and the count data were evaluated as percentages. Continuous variables were expressed by Student’s t-test and Mann-Whitney U-test. Categorical variables were expressed by χ2 or Fisher’s exact test. Statistical significance was taken to be P < 0.05. Univariate logistic regression analyses were used for risk factor. To further analyse the independent risk factors, all statistically significant variables were included in the multivariate model.

## Results

### Clinical characteristics of CRKP strains

In total, 69 non-duplicated isolates were collected from patients recorded with CR-KP infections during the period July 2021-May 2022. The main source of isolates was respiratory tract (41,59.4%), while other sources of isolates included urine(10,14.5%), secretion(6,8.7%), blood(4,5.8%), drainage(2,2.9%), and pus specimen(2,2.9%), etc. The CR-KP strains were divided into CR-hvKP and CR-non-hvKP based on the presence of some combination of *rmpA* and/or *rmpA2* with *iucA*, *iroB* and *peg-344*. PCR analysis revealed 27 (39.1%) strains in the CR-hvKP group, with the remaining 42 (60.9%) strains defined as CR-non-hvKP (Table 1).

Detailed demographic information and the clinical factors of the patients are shown in Table 1. None of the following demonstrated a significant difference between CR-hvKP and CR-non-hvKP: age, gender, admission temperature, admission to ICU, hospitalisation, department, length of stay, underlying diseases, surgery and antibiotic exposure. Furthermore, most invasive procedures and infection types – urinary catheter, central venous catheter, stomach tubes, drainage tube, bone marrow biopsy, pneumonia, urinary infection and bacteremia – did not show a significant difference; the exceptions were tracheal intubation and intracranial infection (74.1 vs. 40.5% and 18.5 vs. 2.4% [CR-hvKP vs. CR-non-hvKP], P = 0.008 and P = 0.020, respectively) (Table 1).

### Antimicrobial susceptibility results

Twenty-one antibiotics were used for the antimicrobial susceptibility testing of 69 CR-KP isolates; these profiles are shown in Table S2. All CR-KP strains were found to be resistant to levofloxacin, cefazolin, ceftriaxone, amoxicillin-clavulanic acid, ertapenem, imipenem, ciprofloxacin, cefuroxime, meropenem, ceftazidime and piperacillin. They showed high resistance to piperacillin-tazobactam, cefoxitin, cefepime, aztreonam, amikacin, gentamicin, tobramycin, sulfamethoxazole and cefperazone-sulbactam but less resistance to ceftazidime/avibactam. There was no statistical significance in the resistance rates of antimicrobial agents between the CR-hvKP and CR-non-hvKP groups except for amikacin (88.90 vs. 61.90%, P = 0.014) (Table S2).

### Multi-locus sequence typing (MLST)

MLST analysis revealed that the 69 CR-KP strains belonged to 14 different STs, among which ST11 was the most prevalent, accounting for 45 (65.2%) of the CR-KP strains. In the CR-hvKP group, there were only three STs: ST11, ST65 and ST16, while the CR-non-hvKP group included a few other rare STs besides these: ST307, ST967, ST37, ST15, ST782, ST219, ST340, ST883, ST656, ST2823 and ST4870. Interestingly, ST65 was only observed in the CR-hvKP group, and ST307 was only observed in the CR-non-hvKP group (Table 1). The detection rates of ST11 and ST65 were significantly higher in the CR-hvKP group than in the CR-non-hvKP group (81.50 vs. 57.10%, P = 0.020 and 3 vs. 0%, P = 0.030, respectively), whereas the proportion of ST307 (0 vs. 16.70%, P = 0.030) was lower.

### Carbapenemase and virulence-associated genes

According to the detection of carbapenemase genes results (Table 1), 73.90% (51/69) of the CR-KP strains carried the *bla*_*kpc−2*_ gene, and this was the dominant carbapenemase gene in both the CR-hvKP and CR-non-hvKP groups (81.50%, 22/27 and 69.00%, 29/42, respectively). In addition, one strain carrying *bla*_*NDM−1*_ and one strain carrying *bla*_*OXA−48*_ were also detected in the CR-hvKP group, while five strains carrying *bla*_*NDM−1*_, three strains carrying *bla*_*NDM−5*_ and one strain carrying *bla*_*OXA−48*_ were also detected in the CR-non-hvKP group. Neither *bla*_*VIM*_ nor *bla*_*IMP*_ were detected in any of the strains, while the differences between the two groups in the detection rates of *bla*_*kpc−2*_ and *bla*_*NDM*_ were not statistically significant.

**Fig. 1:**
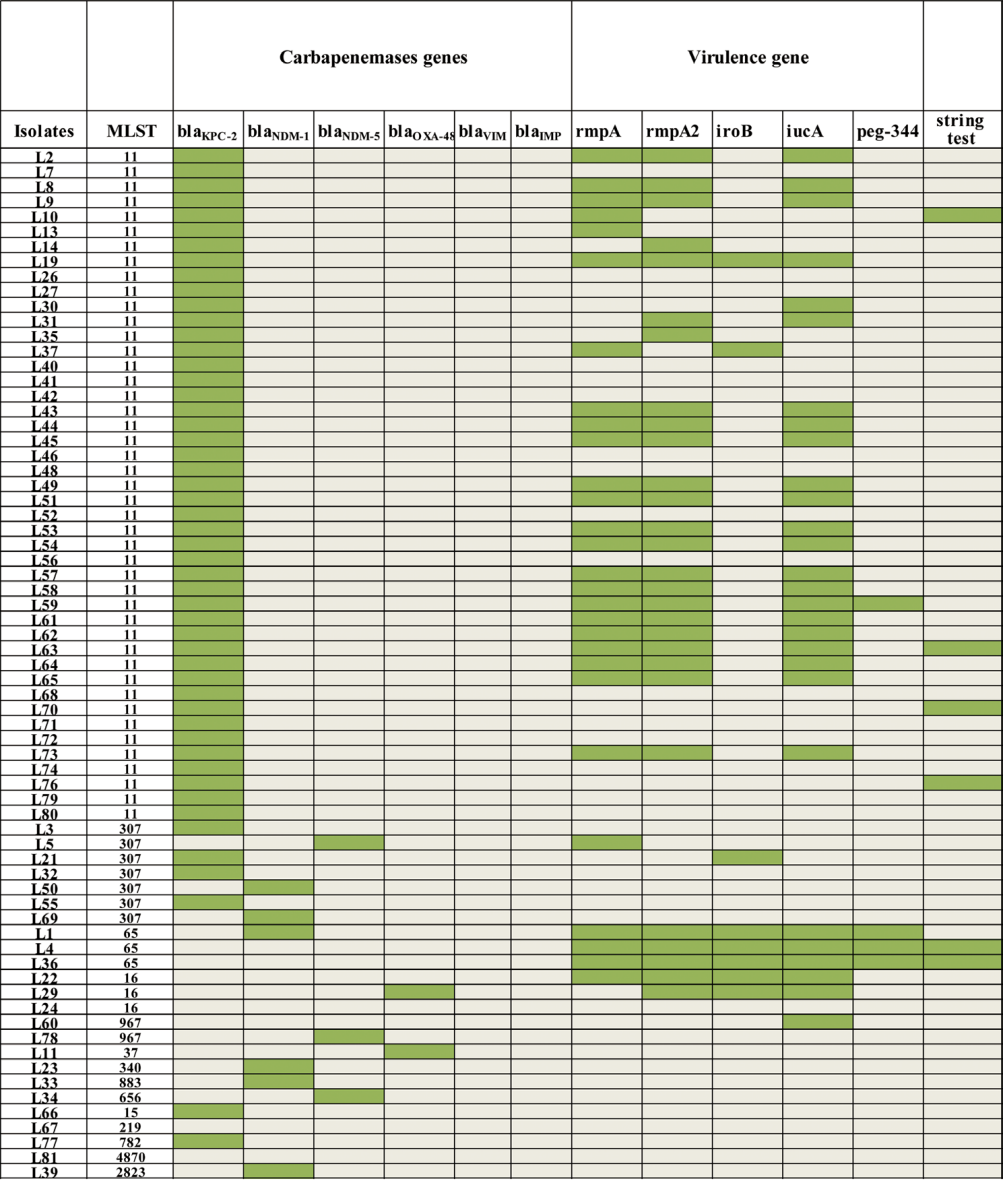
The MLST, string test, virulence genes and carbapenemase genes are shown. The presence of genes is represented by the green box and the absence of genes is represented by the light gray box. Each row of the heatmap (middle) represent a strain

Five virulence genes were detected among the 69 CR-KP strains: *iucA* (40.60%, 28/69), *rmpA* (40.60%, 28/69), *rmpA2* (40.60%, 28/69), *peg-344* (5.80%, 4/69) and *iroB* (10.10%, 7/69). The most prevalent combination was *rmpA* + *rmpA*2 + *iucA* (26.09%, 18/69), followed by *rmpA* + *rmpA*2 + *iroB* + *iucA* + *peg-344* (4.35%, 3/69), *rmpA* + *rmpA*2 + *iroB* + *iucA* (2.90%, 2/69), *rmpA2* + *iucA* (2.90%, 2/69), *rmpA* + *rmpA*2 + *iucA* + *peg-344* (1.45%, 1/69) and *rmpA* + *iroB* (1.45%, 1/69). Moreover, positive results were shown in the string test by 11.1%(3/27)and 7.1% (3/42) of the CR-hvKP and CR-non-hvKP strains, respectively (Fig. 1).

**Table 1:** Microbiological and clinical characteristics of CR-hvKP strains

Factors	CR-KP(n = 69)n(%)	CR-hvKP(n = 27)n(%)	CR-non-hvKP(n = 42)n(%)	*P*-value
**carbapenemases genes**				
KPC-2	51(73.9)	22(81.5)	29(69.0)	0.250
NDM-1	6(8.7)	1(3.7)	5(11.9)	0.240
NDM-5	3(4.3)	0	3(7.10)	0.160
OXA-48	2(2.9)	1(3.7)	1(2.4)	0.790
**MLST**				
ST11	45(65.2)	22(81.5)	23(54.8)	**0.020** ^b^
ST307	7(10.1)	0	7(16.7)	**0.030** ^b^
ST65	3(4.3)	3(11.1)	0	**0.030** ^b^
ST16	3(4.3)	2(7.4)	1(2.4)	0.310
ST967	2(2.9)	0	2(4.7)	0.250
ST37	1(1.4)	0	1(2.4)	0.420
ST15	1(1.4)	0	1(2.4)	0.420
ST782	1(1.4)	0	1(2.4)	0.420
ST219	1(1.4)	0	1(2.4)	0.420
ST340	1(1.4)	0	1(2.4)	0.420
ST883	1(1.4)	0	1(2.4)	0.420
ST656	1(1.4)	0	1(2.4)	0.420
ST2823	1(1.4)	0	1(2.4)	0.420
ST4870	1(1.4)	0	1(2.4)	0.420
Hypermucoviscosity	6(8.7)	3(11.1)	3(7.1)	0.660
KPC-2 ST11	41(59.4)	21(72.4)	20(47.6)	**0.0130** ^b^
**Basic data**				
Age^a^	58 ± 22	57 ± 14	60 ± 16	0.668
Male	54(78.3)	21(77.8)	33(78.6)	0.940
Previous Hospitalizations	40(58.0)	14(51.9)	26(67.0)	0.410
admission to ICU	49(71.0)	21(77.8)	28(66.7)	0.320
Length of stay in hospital ^a^, days	32 ± 41	35 ± 44	30.5 ± 45.3	0.118
Admission temperature ^a^(℃)	37.6 ± 1.1	37.9 ± 1.2	37.5 ± 1.0	0.124
**Department**				
ICU	18(26.1)	7(25.9)	11(26.2)	0.980
Respiratory medicine	11(15.9)	5(18.5)	6(14.3)	0.640
rehabilitation medicine	12(17.4)	4(14.8)	8(19.0)	0.650
Other	28(40.6)	11(40.7)	17(40.5)	0.980
**Underlying diseases**				
Diabetes	16(23.2)	5(18.5)	11(26.2)	0.461
Hypertension	36(52.2)	13(48.1)	23(54.8)	0.590
Cardiovascular disease	38(55.1)	17(63.0)	21(50.0)	0.290
Pulmonary disease	60(87.0)	24(88.9)	34(81.0)	0.380
Hepatobiliary and	38(55.1)	15(55.6)	23(54.8)	0.950
Pancreatic Diseases				
Cerebrovascular disease	37(53.6)	16(59.3)	18(45.2)	0.103
Kidney diseases	43(62.3)	16(59.3)	27(64.3)	0.670
Hematological diseases	31(44.9)	10(37.0)	21(50.0)	0.290
Malignant tumors	10(14.5)	2(7.4)	8(19.0)	0.180
**Infection type**				
Pneumonia	51(73.9)	21(77.8)	30(71.4)	0.560
Urinary infection	9(13.0)	1(3.7)	8(19.0)	0.051
intracranial infection	6(8.7)	5(18.5)	1(2.4)	**0.020** ^b^
Bacteremia	12(17.4)	4(14.8)	8(19.0)	0.650
**Invasive procedures and devices**				
tracheal intubation	37(53.6)	20(74.1)	17(40.5)	**0.008** ^b^
Urinary catheter	51(73.9)	21(77.8)	30(71.4)	0.560
Central intravenous catheter	47(68.1)	21(77.8)	26(67.0)	0.170
Stomach tube	45(65.2)	20(74.1)	25(60.0)	0.210
Drainage tube	17(24.6)	6(22.2)	11(26.2)	0.710
Surgery	28(40.6)	13(48.1)	15(35.7)	0.310
Bone marrow biopsy	8(11.6)	4(14.8)	4(9.5)	0.500
**Antibiotic exposure**				
Cephalosporins	17(24.6)	4(14.8)	13(31.0)	0.130
Carbapenem antibiotic	41(59.4)	16(59.3)	25(60.0)	0.980
β-lactam-β-lactamase	57(82.6)	23(85.2)	34(81.0)	0.650
inhibitors				
Fluoroquinolones	19(27.5)	5(18.5)	14(33.3)	0.180
Aminoglycosides	10(14.5)	4(14.8)	6(14.3)	0.950
Fosfomycin	5(7.2)	1(3.7)	4(9.5)	0.360
Glycopeptides	13(18.8)	4(14.8)	9(21.4)	0.490
Chemotherapy	14(20.3)	8(29.6)	6(14.3)	0.120
**Outcomes**				
Positive outcome	39(56.5)	17(63.0)	22(52.3)	0.390
Negative outcome	30(43.5)	10(37.0)	20(47.6)	0.390

If not otherwise stated, data are reported using frequency and percentage

^a^ Age, admission temperature and length of stay in hospital as mean and standard deviation (SD)

^b^ Bold font means p < 0.05

CR-hvKP, carbapenem-resistant hypervirulent Klebsiella pneumoniae; MLST, multilocus sequence type; ST, Sequence Type

### Risk factors of CR-HvKP infection

Univariate analyses showed that tracheal intubation (P = 0.008), intracranial infection (P = 0.020), neutrophil count (P = 0.049), ST11 (P = 0.020), ST307 (P = 0.030), ST65 (P = 0.030) and *KPC-2* ST11 (P = 0.010) were notable risk factors for CR-HvKP infection. All these variables were included in the multivariate model, and the multivariate logistic regression analysis showed that tracheal intubation (odds ratio [OR] = 4.248; P = 0.030) was an independent risk factor for CR-HvKP infection (Table 2).

**Table 2:** Risk factors for CR-hvKP infections

Variable	Univariate OR (95% CI)	*P*-value	Multivariate OR (95% CI)	*P*-value
intracranial infection	9.318(1.024–84.826)	0.020^a^		
tracheal intubation	4.034(1.395–11.661)	**0.010** ^a^	4.248(1.151–15.682)	**0.030** ^a^
ST11	3.500(1.176–10.414)	0.020^a^		
ST307	0.833(0.728–0.954)	0.030^a^		
ST65	1.125(0.985–1.285)	0.030^a^		
KPC-2 ST11	3.850(1.293–11.640)	0.010^a^		

^a^ Bold font means p < 0.05; ST, Sequence type; OR, Odds Ratio

## Discussion

To our knowledge, this is the first systematic study focusing on CR-hvKP in South China. In recent years, CR-hvKP has been reported to be increasing [12, 13, 22]. CR-hvKP can pose a substantial threat to human health due to its combination of hypervirulent, multidrug resistant and high transmissibility [6, 22, 23]. In order to understand the difference between CR-hvKP and CR-non-hvKP, we investigated and compared the clinical and microbiological characteristics of CR-KP isolates. The results demonstrate that in the First Affiliated Hospital of Guangxi Medical University between July 2021 and May 2022, the dominant *KPC-2*-producing CR-hvKP belonged to ST11. This finding is in general agreement with the fact that CR-hvKP is transmitted in hospitals [22, 24], and suggests that CR-hvKP has become an important and threatening part in cases of CRKP infection in China.

Since 1986, when KP liver abscess complicated by septic endophthalmitis was first reported [25], hvKP has been regarded as the predominant cause of pyogenic liver abscess [26]. In this study, however, patients with CR-hvKP mainly had pneumonia (77.8%) and intracranial infection (18.5%), which is consistent with the conclusions of previous reports [27], and only one patient had an infection that was symptomatic of a liver abscess. Interestingly, intracranial infection was significantly higher in the CR-hvKP group than in the CR-non-hvKP group (P = 0.020), suggesting that in cases of intracranial infection, one should be alert to whether it has been caused by a CR-hvKP strain. Tracheal intubation was also significantly higher in the CR-hvKP than CR-non-hvKP group (P = 0.008), suggesting that tracheal intubation is more likely to lead to CR-hvKP infection. CR-hvKP infection could differ from CR-non-hvKP infection in surveillance for occult infection, source control and site-specific antimicrobial therapy. It is necessary to take preventive and control measures to prevent and treat CR-hvKP infection as early as possible, when the CR-KP strain is cultured by the patient’s cerebrospinal or bronchoalveolar lavage fluid.

Our study showed that *bla*_*kpc−2*_ was the most prevalent carbapenemase gene in the CR-KP isolates, which is consistent with previous studies [28], while CR-hvKP carried one *bla*_*NDM−1*_ and one *bla*_*OXA−48*_, and five, three and one CR-non-hvKP strains harboured *bla*_*NDM−1*_, *bla*_*NDM−5*_ and *bla*_*OXA−48*_, respectively. In addition, *bla*_*kpc−2*_, *bla*_*NDM−1*_, *bla*_*NDM−5*_, *bla*_*OXA−48*_, *bla*_*VIM*_ and *bla*_*IMP*_ were not detected in seven strains, this may be indicitive of other mechanisms involved in carbapenem resistance, such as efflux pumps and porin mutations [29].

Due to express carbapenemase and extended-spectrum β-lactamase, CR-KP strains are resistant to most general antibacterial drugs [30]. Here, the antimicrobial susceptibility testing showed that all the CR-KP strains were highly or completely resistant to general antibacterial drugs, such as piperacillin-tazobactam, cefoxitin, cefepime, aztreonam, amikacin, gentamicin, tobramycin, sulfamethoxazole and cefperazone-sulbactam, while the resistance rate to ceftazidime/avibactam was relatively low, accounting for 26.1%, which is higher than the results of Zhou et al. [31]. As reported in previous studies, colistin and tigecycline were still a good choice for the treatment of CR-KP infection [32, 33]. However, the side effects of these drugs should also be taken seriously, and they should be used with caution. Regardless of whether a CR-KP infection is hvKP or non-hvKP, combination susceptibility testing should be prioritized to determine the appropriate antibiotic combination.

In our study, the most dominant sequence type of the 69 CR-KP isolates was ST11 (65.2%, 42/69), which is consistent with a previous conclusion that this is the most common type of CR-KP in Western China [34]. Further, the detection rates of ST11 and ST65 were significantly higher in the CR-hvKP strains than in the CR-non-hvKP strains, while ST307 was significantly lower. Of the 29 CR-hvKP strains, 22 (75.9%) were ST11, which is consistent with the ST11 CR-hvKP finding described by Gu et al. [6]. According to one previous study, 80% (16/20) of KP isolates included hvKP strains belonging to clones ST23 and ST65 [35]. However, no ST23 strain was found in either of the CR-KP groups here, and only three ST65 strains were found in the CR-hvKP group. Interestingly, one of these three causes pyogenic liver abscess. To the best of our knowledge, the report about ST65 CR-hvKP was unusual; we should be alert to its prevalence.

To date, no consensus definition has emerged for CR-hvKP, and the microbiological features of CR-hvKP vary from study to study. Some previous studies have shown that most CR-hvKP strains were positive in string tests [6, 36], but the results of our study revealed that only three of the 27 (11.1%) CR-hvKP and three of the 42 (7.1%) CR-non-hvKP isolates were positive. Therefore, the string test showed suboptimal identification accuracy for CR-hvKP. Interestingly, the string test was used for the identification of a hypermucoviscous phenotype regulated primarily by *rmpA* or *rmpA2*, however, two of the six positive string test strains did not bring *rmpA* or *rmpA2*. This indicates that KP which exhibits hypermucoviscosity and yet does not harbor *rmpA* or *rmpA2* has already appeared in clinical settings [37].

In this study, the identification of CR-hvKP was based on the presence of any combination of the virulence genes *rmpA* and/or *rmpA2* with *iucA*, *iroB*, or *peg-344*. These markers were found to be highly predictive for hvKp. Their combinations were *rmpA* + *rmpA*2 + *iucA*, *rmpA* + *rmpA*2 + *iroB* + *iucA* + *peg-344*, *rmpA* + *rmpA*2 + *iroB* + *iucA*, *rmpA2* + *iucA*, *rmpA* + *rmpA*2 + *iucA* + *peg-344* and *rmpA* + *iroB*. We found that all combinations of the five virulence genes contained one or both of *rmpA* and *rmpA2*, which suggested that these two virulence genes might be indispensable for the definition of CR-hvKP. Furthermore, only three CR-hvKP strains carried all five of the virulence genes and these three strains were all ST65, which suggested they might harbour the full length of the virulence plasmid pNTUH-K2044, pLVPK and pLVPK-like. Although the five biomarkers used have a high diagnostic accuracy for identifying hvKP, it is not known which combination best predicts CR-hvKP or even which experimental combinations should be used to improve accuracy. Thus, the international criteria defining CR-hvKP require further study.

Our study has shown the prevalence of CR-hvKP infection to be 39.1%. The vast majority of these strains were *KPC-2*-producing and ST11, which is consistent with previous research [22, 38]. The *KPC-2* ST11 clone has been determined as the most predominant genotype of CR-KP in China [39–41], but it was significantly higher here in the CR-hvKP group than in the CR-non-hvKP group (P = 0.013), which showed that the spread of virulence genes in this clone is of particular concern. Therefore, a better understanding of the risk factors of CR-hvKP infection is essential for intervention.

Our results have shown that tracheal intubation is an independent risk factor for CR-hvKP infection (P = 0.030, OR = 4.131), which indicates that appropriate intervention measures to prevent infection should be taken. Due to the greater virulence of CR-hvKP, the medication and management of CR-hvKP infection are different from those of CR-non-hvKP infection; clinicians should pay more attention to this risk factor in clinical practice to prevent and prevent the spread of CR-hvKP strains.

This study had some limitations. First, it was an 11-month retrospective study conducted at a single centre rather than a multicentre epidemiological study of CR-hvKP, and the number of patients was small. Second, we did not perform antimicrobial susceptibility testing of tigecycline and polymyxin and cannot know whether the rates of resistance to these two antimicrobials were consistent with previous research. Third, although the five virulence genes *rmpA*, *rmpA2*, *iucA*, *iroB* and *peg-344* can be used to predict hv phenotype, they still do not reflect the actual virulence of KP. Preferably, in order to identify the hvKP strain, in vivo and in vitro experiments should be performed involving, for example, galleria model, mouse model, human neutrophil experiment and whole genome sequencing.

## Conclusions

The prevalence of CR-hvKP may be higher than expected in the healthcare setting. CR-hvKP is gradually becoming the dominant nosocomial pathogen. Here, tracheal intubation has been found to be an independent variable for CR-hvKP infection. According to the clinical data we collected, this procedure was performed mainly in the elderly with severe underlying diseases. We speculate that CR-hvKP has become prevalent in older adults with comorbidities in hospitals. The prevalence and treatment of CR-hvKP will present a major challenge. It is essential to enhance the clinical awareness and management of CR-hvKP infection, especially among elderly patients.

### Electronic supplementary material

Below is the link to the electronic supplementary material.

Supplementary Material 1

## Data Availability

The datasets used and/or analysed during the current study are available from the corresponding author upon reasonable request.
