# Supplementary material for: Infection with Carbapenem-resistant Hypervirulent Klebsiella Pneumoniae: clinical, virulence and molecular epidemiological characteristics
Source: Antimicrob Resist Infect Control. 2023 Nov 13;12:124. doi: 10.1186/s13756-023-01331-y (PMC10642049; doi:10.1186/s13756-023-01331-y)
Supplement: Supplementary file 1 — Supplementary Material 1 [file 13756_2023_1331_MOESM1_ESM.docx]

**Infection with Carbapenem-resistant Hypervirulent Klebsiella Pneumoniae: Clinical, Virulence and Molecular Epidemiological Characteristics**

**TABIE S1: Primers used in this study.**

| Genes | Primer sequence (5′-3′) | Annealing temperature(◦C) | Size (bp) |
| --- | --- | --- | --- |
| **Virulence genes** |  |  |  |
| rmpA | F:ATGTGGCTTGACGTTTCGGGGG | 55 | 160 |
|  | R:GCCGTGGATAATGGTTTACAATTCGGC |  |  |
| rmpA2 | F:GGATGTGGCTTGACATTTCGGGGG | 55 | 227 |
|  | R:TTCATGGATGCCCTCCCTCCTG |  |  |
| iroB | F:ATCTCATCATCTACCCTCCGCTC | 58 |  |
|  | R:GGTTCGCCGTCGTTTTCAA |  |  |
| iucA | F:AATCAATGGCTATTCCCGCTG | 56 | 239 |
|  | R:CGCTTCACTTCTTTCACTGACAGG |  |  |
| peg-344 | F:CTTGAAACTATCCCTCCCTCCAGTC | 55 | 508 |
|  | R:CCAGCGAAAGAATAACCCC |  |  |
| **Carbapenemase genes** |  |  |  |
| KPC | F:ATGTCACTGTATCGCCGTCT | 55 | 893 |
|  | R:TTTTCAGAGCCTTACTGCCC |  |  |
| NDM | F:ATGGAATTGCCCAATATTATGC | 55 | 813 |
|  | R:TCAGCGCAGCTTGTCGG |  |  |
| 0XA-48 | F:GCGTGGTTAAGGATGAACAC | 56 | 438 |
|  | R:CATCAAGTTCAACCCAACCG |  |  |
| VIM | F:GGTCGCATATCGCAACGCAGT | 55 | 636 |
|  | R:CGGCGACTGAGCGATTTTTG |  |  |
| IMP | F:ATGAGCAAGTTATCTGTATTCTTTAT | 55 |  |
|  | R:TTAGTTGCTTAGTTTTGATGGTTT |  | 741 |
| **Housekeeping genes** |  |  |  |
| rpoB | F:GGCGAAATGGCWGAGAACCA | 50 | 501 |
|  | R:GAGTCTTCGAAGTTGTAACC |  |  |
| gapA | F:TGAAATATGACTCCACTCACGG | 60 | 450 |
|  | R:CTTCAGAAGCGGCTTTGATGGCTT |  |  |
| mdh | F:CCCAACTCGCTTCAGGTTCAG | 50 | 477 |
|  | R:CCGTTTTTCCCCAGCAGCAG |  |  |
| pgi | F:GAGAAAAACCTGCCTGTACTGCTGGC | 50 | 432 |
|  | R:CGCGCCACGCTTTATAGCGGTTAAT |  |  |
| phoE | F:ACCTACCGCAACACCGACTTCTTCGG | 50 | 420 |
|  | R:TGATCAGAACTGGTAGGTGAT |  |  |
| infB | F:CTCGCTGCTGGACTATATTCG | 50 | 318 |
|  | R:CGCTTTCAGCTCAAGAACTTC |  |  |
| tonB | F:CTTTATACCTCGGTACATCAGGTT | 45 | 414 |
|  | R:ATTCGCCGGCTGRGCRGAGAG |  |  |

TABIE S2: Susceptibility of CR-hvKP and CR-non-hvKP strains against different antimicrobial agents.

| **Antibiotic agent** | **CR-KP(n=69)n%** | **CR-hvKP（n=27)n%** | **CR-non-hvKP(n=42)n%** | ***P*-value** |
| --- | --- | --- | --- | --- |
| ceftazidime/avibactam | 18（26.1） | 5(18.5) | 13(31.0) | 0.179 |
| piperacillin-tazobactam | 68（98.6） | 26(96.3) | 42(100.0) | 0.209 |
| cefepime | 66（95.7） | 26(96.3) | 40(95.2) | 0.833 |
| cefoxitin | 68（98.6） | 27(100.0） | 41(97.6) | 0.419 |
| aztreonam | 67（97.1） | 27(100.0） | 40(95.2) | 0.250 |
| amikacin | 50（72.5） | 24(88.9) | 26(61.9) | **0.014** |
| gentamicin | 59（85.5） | 24(88.9) | 35(83.3) | 0.522 |
| tobramycin | 62（89.9） | 25(92.6) | 37(88.1) | 0.546 |
| sulfamethoxazole | 51(73.9) | 23(85.2) | 28（66.7） | 0.807 |
| Cefperazone-Sulbactam | 68(98.6) | 26(96.3) | 42（100.0） | 0.209 |
| levofloxacin | 69（100.0） | 27（100.0） | 42（100.0） | / |
| cefazolin | 69（100.0） | 27（100.0） | 42（100.0） | / |
| ceftriaxone | 69（100.0） | 27（100.0） | 42（100.0） | / |
| amoxicillin-clavulanic acid | 69（100.0） | 27（100.0） | 42（100.0） | / |
| ertapenem | 69（100.0） | 27（100.0） | 42（100.0） | / |
| imipenem | 69（100.0） | 27（100.0） | 42（100.0） | / |
| ciprofloxacin | 69（100.0） | 27（100.0） | 42（100.0） | / |
| cefuroxime | 69（100.0） | 27（100.0） | 42（100.0） | / |
| meropenem | 69（100.0） | 27（100.0） | 42（100.0） | / |
| ceftazidime | 69（100.0） | 27（100.0） | 42（100.0） | / |
| piperacillin | 69（100.0） | 27（100.0） | 42（100.0） | / |
